# Structural diversity and phylogenomic insights from the mitochondrial genomes of two *populus* species from the Qinghai-Tibet Plateau

**DOI:** 10.3389/fpls.2025.1637726

**Published:** 2025-09-12

**Authors:** Jin-Liang Huang, Yu-Jie Shi, Fei-Fei Tian, Jia-Xuan Mi, Fan Zhang, Shi-Xing Zhou, Xue-Qin Wan, Cong-De Huang

**Affiliations:** ^1^ College of Forestry, Sichuan Agricultural University, Chengdu, China; ^2^ College of Life Sciences, Zhejiang Provincial Key Laboratory of Plant Evolutionary Ecology and Conservation, Taizhou University, Taizhou, China; ^3^ College of Forestry, Southwest Forestry University, Kunming, China; ^4^ College of Landscape Architecture, Sichuan Agricultural University, Chengdu, China; ^5^ Sichuan Province Key Laboratory of Ecological Forestry Engineering on the Upper Reaches of the Yangtze River, Ya’an, Sichuan, China; ^6^ Sichuan Mt. Emei Forest Ecosystem National Observation and Research Station, Sichuan Agricultural University, Chengdu, China

**Keywords:** *Populus*, mitochondrial genome, phylogenetic reconstruction, Qinghai –Tibet Plateau, adaptive evolution

## Abstract

*Populus kangdingensis* and *P. ciliata* are important tree species distributed across the Qinghai–Tibet Plateau, yet the structure and evolutionary characteristics of their mitochondrial genomes remain unclear. To elucidate these features and their phylogenetic relationships, we assembled the mitochondrial genomes of these two species using PacBio HiFi sequencing data with PMAT2, and annotated them with PMGA. The results showed that the mitochondrial genome of *P. kangdingensis* consists of three independent circular molecules with a total length of 785,824 bp, while that of *P. ciliata* exhibits a branched structure comprising two circular molecules and one linear molecule, totaling 798,807 bp. Both genomes contain 57 functional genes, including 34 highly conserved protein-coding genes. Comparative genomic analyses revealed differences in repeat sequences, RNA editing patterns, and chloroplast-derived sequences, suggesting species-specific evolution at the organellar genome level. Ka/Ks analysis identified genes such as atp4, ccmB, and mttB as potentially under positive selection, reflecting adaptation to high-altitude environments. Phylogenetic trees constructed using 30 mitochondrial and 58 chloroplast protein-coding genes confirmed the monophyly of Populus and Salix, and identified them as sister groups. However, topological inconsistencies observed within Populus suggest the influence of lineage sorting, hybridization, and differences in mutation rates. This study provides the first high-quality mitochondrial genomes of *P. kangdingensis* and *P. ciliata*, revealing the structural diversity of multi-circular and branched conformations in Populus mitochondrial genomes, and offering new insights into organellar genome evolution and high-altitude adaptation in this genus.

## 1 Introduction

Mitochondria are essential organelles in eukaryotic cells responsible for aerobic respiration and energy production. Beyond generating ATP through oxidative phosphorylation, they are also involved in critical physiological processes such as programmed cell death, metabolic regulation, cytoplasmic male sterility, and signal transduction (Chandel, 2014; Kim and Zhang, 2018). Compared with animal mitochondria, plant mitogenomes exhibit remarkable structural diversity and evolutionary complexity, characterized by large genome sizes, low coding density, frequent structural rearrangements, abundant repetitive sequences, and extensive incorporation of chloroplast and nuclear DNA fragments (Sloan et al., 2012; Petersen et al., 2015; Wynn and Christensen, 2019). Traditionally, plant mitochondrial genomes have been portrayed as single circular molecules. However, with the advent of high-throughput and long-read sequencing technologies, an increasing number of species have been found to possess multipartite, linear, or branched mitochondrial genome conformations (Alverson et al., 2011; Burger et al., 2012). These structural variations highlight the highly dynamic nature of plant mitochondrial genomes and provide important clues for exploring recombination mechanisms, replication regulation, and species evolution (Palmer et al., 2000; Rice et al., 2013; Sanchez-Puerta et al., 2017).

*Populus* is a genus of fast-growing deciduous trees with significant ecological, breeding, and molecular research value (Tuskan et al., 2006). In recent years, mitogenomes of several *Populus* species have been reported, including *P. tremula*, *P. alba*, *P. davidiana*, *P. adenopoda*, and *P. simonii*, with genome sizes ranging from 772 to 869 kb. These genomes typically contain over 30 protein-coding genes (PCGs), more than 20 tRNA genes, and 3 rRNA genes. Previous genome assemblies based on next-generation and long-read sequencing platforms, including Illumina, Ion Torrent, and PacBio—such as those for *P. tremula* (Kersten et al., 2016), *P. alba* (Brenner et al., 2019), and *P. davidiana* (Choi et al., 2017)—revealed a single linear mitochondrial genome structure, likely due to the relatively low coverage of long-read sequencing. In contrast, more recent assemblies using third-generation sequencing data, including those of *P. trichocarpa*, *P. simonii* (Bi et al., 2022), and *P. deltoides* (Qu et al., 2023), have revealed multipartite mitochondrial genomes, challenging the previously resolved structures. The species *P. kangdingensis* and *P. ciliata* are important montane *Populus* species native to the Qinghai–Tibet Plateau, exhibiting notable adaptations to extreme high-altitude environments such as low oxygen and high UV radiation (Shi et al., 2023, 2024b). However, to date, there has been no comprehensive study on the mitogenomes of these two species. Given the unique advantages of mitogenomes in maternal inheritance, species differentiation, and phylogenetic reconstruction, sequencing and comparative analysis of the mitogenomes of *P. kangdingensis* and *P. ciliata* will deepen our understanding of their adaptive evolution in plateau environments and fill a knowledge gap in *Populus* mitochondrial genomics.

In this study, we utilized PacBio HiFi long-read sequencing to assemble and annotate high-quality mitogenomes for these two species for the first time. We characterized their structural configurations, number of genes, RNA editing sites, codon usage bias, and repeat sequence distributions, compared them with mitogenomes of eight other *Populus* species, and conducted phylogenetic analyses to infer their evolutionary relationships. This study not only extends the known structural diversity of *Populus* mitogenomes to include linear conformations, but also provides foundational data and theoretical insights for understanding mitogenome evolution, multi-source DNA transfer, and lineage divergence in the genus *Populus*.

## 2 Materials and methods

### 2.1 Sample collection and sequencing data acquisition

Branches of *P. kangdingensis* and *P. ciliata* were collected from the Poplar Germplasm Repository at the College of Forestry, Sichuan Agricultural University (103°38′42″E, 30°33′97″N), and hydroponically acclimated in a controlled climate chamber for six weeks (He et al., 2025). Fresh young leaves were subsequently harvested, flash-frozen in liquid nitrogen, and stored at –80°C until use. Genomic DNA was extracted using a modified CTAB protocol (He et al., 2024), and DNA integrity and purity were assessed with a NanoDrop spectrophotometer and a Qubit v4 fluorometer (Invitrogen). High-quality samples (OD260/280 between 1.8 and 2.0, OD260/230 > 2.0, with distinct main bands and no observable degradation) were selected for library preparation. Single Molecule Real-Time (SMRT) sequencing was performed on the PacBio Sequel II platform. Raw reads were processed to remove adapter sequences and filtered for quality. Circular consensus sequences (HiFi reads) were then generated using the CCS software.

### 2.2 Mitochondrial genome assembly and annotation

The mitochondrial genomes were assembled using PMAT2 (Bi et al., 2024), generating initial assembly graphs. The assembly was visualized using Bandage v0.9.0 (Wick et al., 2015). Contigs suspected to be chloroplast- or nuclear-derived were first identified based on their distinctively higher or lower read coverage in the assembly graph, and then aligned against reference chloroplast sequences for confirmation. These non-mitochondrial contigs were subsequently removed from the assembly. The mitochondrial genome structures were resolved based on assembly graph topology and sequencing read coverage depth, with conformations parsed using Bandage visualization and guided by reference to the *P. trichocarpa* genome. Repeat regions were identified and analyzed, resulting in finalized mitochondrial genome assemblies. Annotation was performed using the Intelligent Plant Mitochondrial Genome Annotator (IPMGA; Li et al., 2025). The Plant Mitochondrial Genome map (PMGmap; Zhang et al., 2024) online tool was used to visualize the genome and generate cis- and trans-splicing gene maps. The mitochondrial genomes of *P. kangdingensis* and *P. ciliata* were then submitted to GenBank to obtain accession numbers.

### 2.3 Comparative analysis of mitochondrial genomes

The mitochondrial genomes of *P. kangdingensis* and *P. ciliata* were compared with those of eight other *Populus* species (*P. trichocarpa*, *P. deltoides, P. alba, P. adenopoda, P. simonii, P. tremula, P. davidiana, and P. rotundifolia*), as listed in **Supplementary Table S1**. All mitochondrial genomes were re-annotated using the same pipeline described in section 2.2 to ensure consistency across species. Genome metrics including total length, GC content, gene counts, and intron structure were calculated using the Genepioneer Cloud platform (http://cloud.genepioneer.com:9929/#/home). Protein-coding genes (PCGs) were extracted using custom Python scripts. The Ka/Ks ratios for 30 conserved mitochondrial PCGs across the 10 *Populus* species were calculated using KaKs_Calculator v2.0 (Wang et al., 2010).

### 2.4 Codon usage bias analysis

PhyloSuite v1.2.3 (Zhang et al., 2020) was used to calculate relative synonymous codon usage (RSCU) values and codon counts for each amino acid across the PCGs of the 10 species. Visualizations were produced via the Genepioneer Cloud. GC content at the first, second, and third codon positions (GC1, GC2, GC3) and the effective number of codons (ENC) were calculated for *P. kangdingensis* and *P. ciliata*. ENC-GC3 plots were used to assess the influence of selection and mutation bias on codon usage.

### 2.5 Repeat sequences and RNA editing site analysis

Simple sequence repeats (SSRs) were identified using the MISA online tool (Beier et al., 2017). Tandem repeats were identified using Tandem Repeat Finder (Benson, 1999), and four types of dispersed repeats were analyzed with REPuter (Kurtz and Schleiermacher, 1999), all using default parameters. C-to-U RNA editing sites in the 34 PCGs of both species were predicted using Deepred-mt (Edera et al., 2021) with default settings.

### 2.6 DNA transfer between mitochondria and chloroplasts

Previously assembled chloroplast genomes of *P. kangdingensis* and *P. ciliata* were used for homology searches against their mitochondrial genomes via BLAST, using an E-value threshold of 1e-5. Homologous segments with significant matches were identified as transferred mitochondrial plastid segments (MtPts). BEDTools v2.28.0 (Quinlan and Hall, 2010) was used to annotate MtPts based on GFF3 files, categorizing gene types and integrity (Complete/Partial). Circos v0.69-9 (Krzywinski et al., 2009) was used to visualize syntenic connections between mitochondrial and chloroplast genomes.

### 2.7 Synteny analysis

To investigate evolutionary relationships within *Populus* and among other genera from Malpighiales, synteny analysis was conducted between *P. kangdingensis* and representative species from six genera in Malpighiales (**Supplementary Table S1**), as well as among *P. kangdingensis*, *P. ciliata*, and previously published species from the sect. *Tacamahaca*, sect. *Aigeiros*, and sect. *Populus* (Zhao et al., 2024). Synteny and visualization were performed using NGenomeSyn v1.42 (He et al., 2023). Pairwise synteny was analyzed using the GetTwoGenomeSyn.pl script with minimap2, followed by manual configuration file preparation for visualization.

### 2.8 Phylogenetic analysis

All available mitochondrial and chloroplast genomes from 11 genera and 35 species in Malpighiales were downloaded from NCBI (**Supplementary Table S1**). Shared PCGs (19 mitochondrial, 58 chloroplast) were extracted using PhyloSuite v1.2.3, and gene trees for each were constructed using FastTree2 (Price et al., 2010). Species trees were then reconstructed using ASTRAL-IV (Zhang and Mirarab, 2022). Mitochondrial and chloroplast phylogenies were visualized using the tvBOT online tool (Xie et al., 2023).

## 3 Results and analysis

### 3.1 Mitochondrial genome features of *P. kangdingensis* and *P. ciliata*

**Figure 1:**
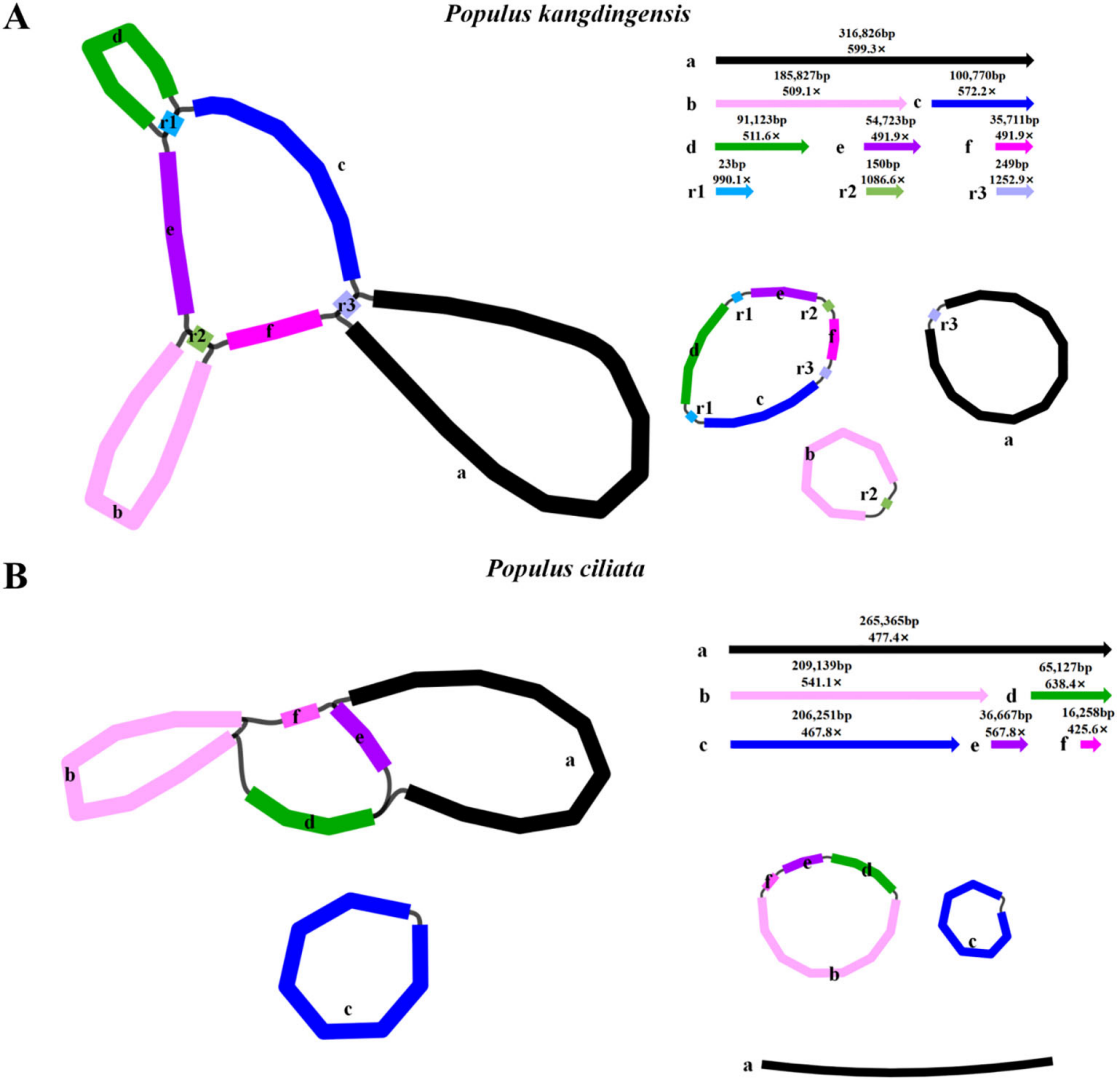
Structure of the mitochondrial genomes of *P. kangdingensis* and *P. ciliata*. **(A)** Circular configuration of the *P. kangdingensis* mitogenome, composed of three independent circular molecules. **(B)** Branched configuration of the *P. ciliata* mitogenome, consisting of two circular molecules and one linear molecule.

HiFi sequencing using the PacBio Sequel II platform generated 93 GB and 39 GB of data for *P. kangdingensis* and *P. ciliata*, respectively. For *P. kangdingensis*, a total of 5,538,529 HiFi reads were obtained, with a maximum length of 59,758 bp and an average length of 16,803 bp. For *P. ciliata*, 2,337,475 HiFi reads were produced, with a maximum length of 51,421 bp and an average length of 17,003 bp. Mitogenomes were assembled using PMAT2 and manually curated. Bandage visualization showed multiple potential conformations in both P*. kangdingensis* and *P. ciliata*. We resolved one conformation by referring to the previously reported *P. trichocarpa* mitochondrial genome.The mitochondrial genome of *P. kangdingensis* consists of three circular molecules (**Figure 1A**), with a total length of 785,824 bp and a GC content of 44.74%. The sizes of the three subgenomes are 317,075 bp, 282,772 bp, and 185,977 bp, respectively (**Supplementary Figure S1A**). In contrast, the mitochondrial genome of *P. ciliata* exhibits a branched structure (**Figure 1B**), comprising two circular molecules and one linear molecule with a total length of 798,807 bp and a GC content of 44.73%. The two circular molecules are 327,191 bp and 206,251 bp in length, and the linear molecule is 265,365 bp (**Supplementary Figure S1B**). Annotation using PMGA identified 57 shared genes in both species, including 34 protein-coding genes (PCGs), 20 tRNA genes, and 3 rRNA genes (**Supplementary Table S2**). Among the PCGs, 24 are core genes and 10 are non-core. The core genes include five ATP synthase genes (*atp1*, *atp4*, *atp6*, *atp8*, *atp9*), four cytochrome c biogenesis genes (*ccmB*, *ccmC*, *ccmFC*, *ccmFN*), one cytochrome c reductase gene (*cob*), three cytochrome c oxidase genes (*cox1*, *cox2*, *cox3*), one maturase (matR), one membrane transporter (mttB), and nine NADH dehydrogenase genes (*nad1, nad2, nad3, nad4, nad4L, nad5, nad6, nad7, nad9*). The non-core genes include three large subunit ribosomal proteins (*rpl2, rpl10, rpl16*), five small subunit ribosomal proteins (*rps1, rps3, rps4, rps7, rps12*), and two succinate dehydrogenase genes (*sdh3, sdh4*). Additionally, four cis-splicing genes (*ccmFC, nad4, nad7, rps3*) and three trans-spliced genes (*nad1, nad2, nad5*) were identified. Details of exons for each gene are provided in **Supplementary Figures S2** and **S3**.

### 3.2 Comparative analysis of mitogenomes in *Populus*

**Figure 2:**
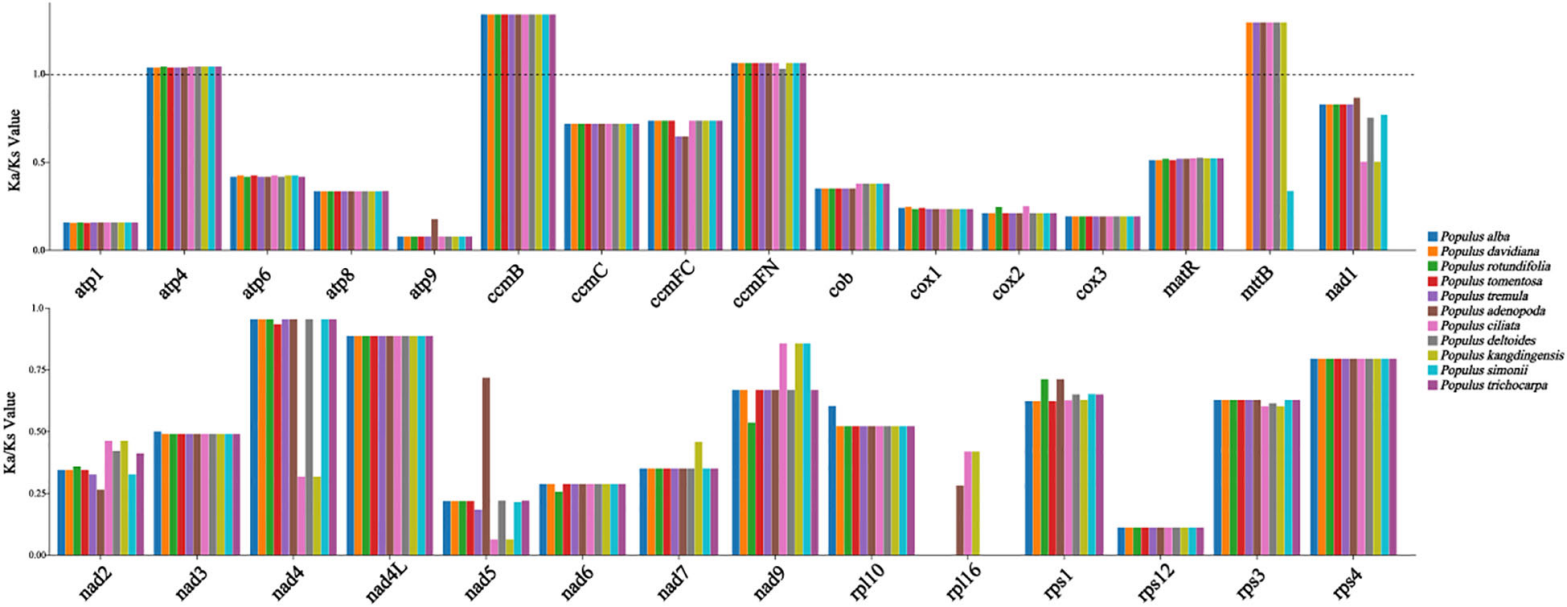
Ka/Ks ratios of 30 shared mitochondrial PCGs across 10 *Populus* taxa. Each bar represents the Ka/Ks ratio of a specific gene in a particular species. Colors denote different species, as indicated in the legend. The dashed line marks the reference value of Ka/Ks=1.

To investigate the evolutionary features of *Populus* mitogenomes, we compared *P. kangdingensis* and *P. ciliata* with eight other species: *P. simonii*, *P. trichocarpa*, *P. adenopoda*, *P. alba*, *P. davidiana*, *P. deltoides*, *P. rotundifolia*, and *P. tremula*. The size of mitogenomes ranged from 772,549 bp (*P. rotundifolia*) to 869,108 bp (*P. adenopoda*), with GC content varying between 44.7% (*P. adenopoda*) and 44.85% (*P. trichocarpa*). Detailed values were listed in **Supplementary Table S3**. *P. kangdingensis* possessed the highest gene count (60), while *P. rotundifolia* had the fewest (54). The number of PCGs was relatively consistent among species, although intron numbers in NADH dehydrogenase genes varied. For instance, *nad1*, *nad4*, and *nad7* in *P. kangdingensis* contained 1, 1, and 4 introns respectively, while in *P. ciliata* the counts were 2, 3, and 3. The genes *rps14* were absent in *P. kangdingensis*, *P. ciliata*, and *P. simonii*, and *sdh3* was only present in *P. kangdingensis*, *P. ciliata*, and *P. adenopoda*. All species had 3 rRNA genes, with most differences arising from the number of tRNA genes. To assess the effect of environmental pressure on mitogenome evolution, we calculated Ka/Ks ratios for 30 shared PCGs across all ten *Populus* species (**Figure 2**). Most genes had Ka/Ks < 1, indicating purifying selection. However, *atp4*, *ccmB*, *ccmFN*, and *mttB* had Ka/Ks > 1 in most species, suggesting positive selection, though in a few species these genes showed signs of purifying selection instead.

### 3.3 Codon usage bias in *Populus* mitochondrial genomes

**Figure 3:**
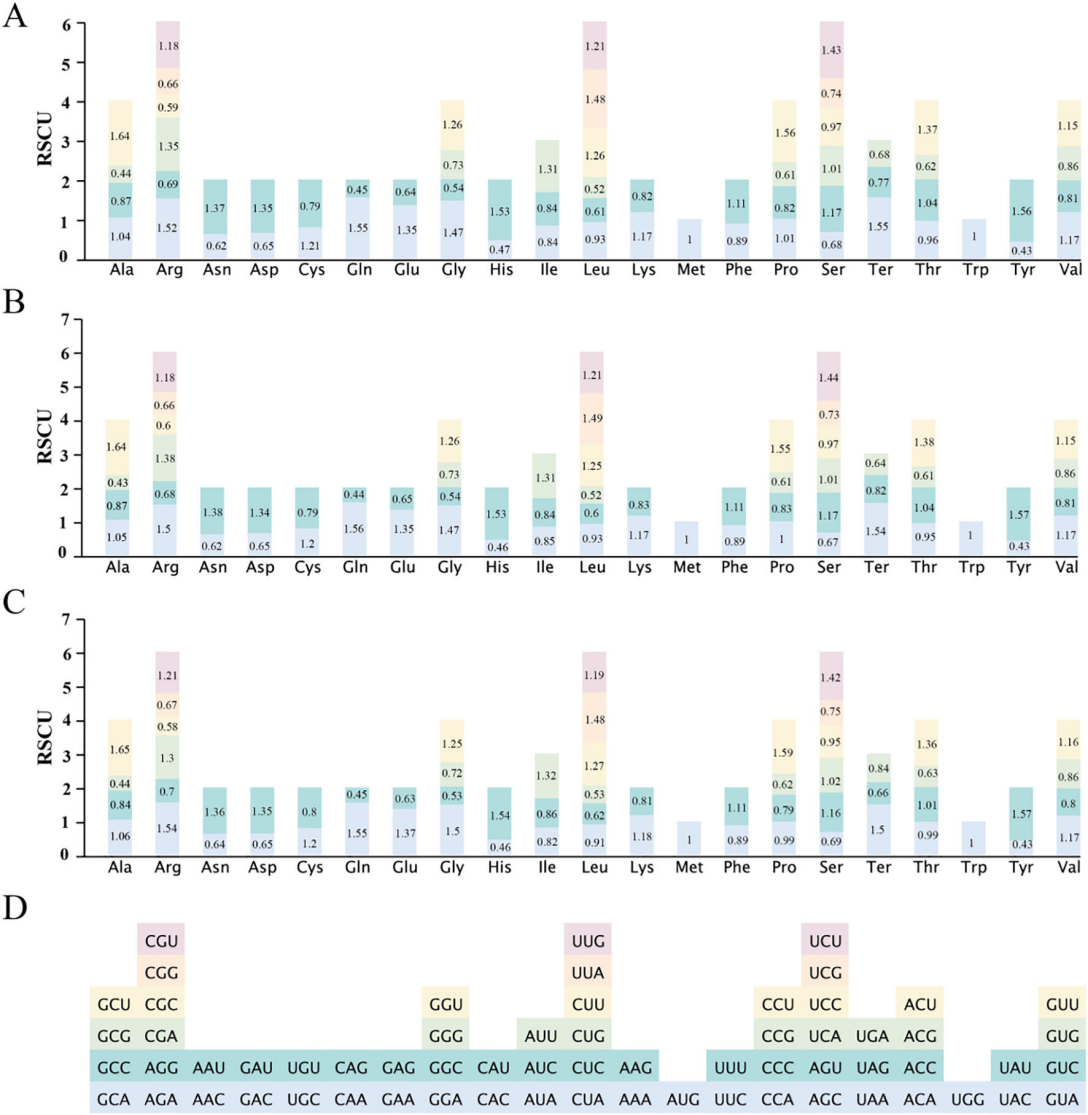
RSCU values of 64 codons in the mitogenomes of ten *Populus* species. **(A)** Mean RSCU values across the ten *Populus* species; **(B)** RSCU values in *P. kangdingensis*; **(C)** RSCU values in *P. ciliata*; **(D)** Codon color legend. Each stacked bar represents RSCU values for synonymous codons corresponding to a specific amino acid. RSCU > 1 indicates higher-than-expected usage; RSCU < 1 indicates lower-than-expected usage. Codons are color-coded and grouped under their respective amino acids.

In the ten *Populus* mitogenomes, codon usage analysis identified 64 codons encoding 21 amino acids (**Figure 3D**). The protein-coding genes (PCGs) comprised a total of 101,650 codons, with species-level counts ranging from 9,831 to 10,871. *P. kangdingensis* contained the fewest codons (9,831), followed by *P. ciliata* (9,851). Among all codons, UUU was the most frequently used across species. Leucine (Leu) and serine (Ser), each encoded by six synonymous codons, were the most abundant amino acids, accounting for 11,592 codons (11.4%) and 9,084 codons (8.94%), respectively. The mean relative synonymous codon usage (RSCU) values across species showed that 30 codons had RSCU > 1, while 32 codons had RSCU < 1. Methionine (AUG) and tryptophan (UGG) exhibited no codon usage bias RSCU = 1 (**Figure 3A**). In *P. kangdingensis*, RSCU patterns were consistent with the overall mean (**Figure 3B**). By contrast, in *P. ciliata*, 30 codons showed RSCU > 1 and 33 codons showed RSCU < 1, with a notable deviation in the CCA codon for proline (Pro) (**Figure 3C**). Collectively, except for AUG and UGG, most amino acids displayed codon usage bias.

To further examine codon usage patterns in *P. kangdingensis* and *P. ciliata*, we analyzed 34 PCGs, calculating GC content at the first (GC1), second (GC2), and third (GC3) codon positions, and the effective number of codons (ENC). GC1 ranged from 36.08% to 56.3%, GC2 from 30.3% to 55.47%, and GC3 from 25.55% to 50.5% (**Supplementary Table S4**), all averaging below 50%, suggesting a preference for A/T-ending codons. ENC values ranged from 28.47 to 57.36, with averages of 52.95 (*P. kangdingensis*) and 52.93 (*P. ciliata*), indicating weak codon usage bias (**Supplementary Figure S4**). ENC-GC3 plots showed that most PCGs deviated from the expected curve, suggesting codon bias is influenced by gene expression levels.

### 3.4 Repeat sequence analysis and RNA editing site prediction in *P. kangdingensis* and *P. ciliata*

**Figure 4:**
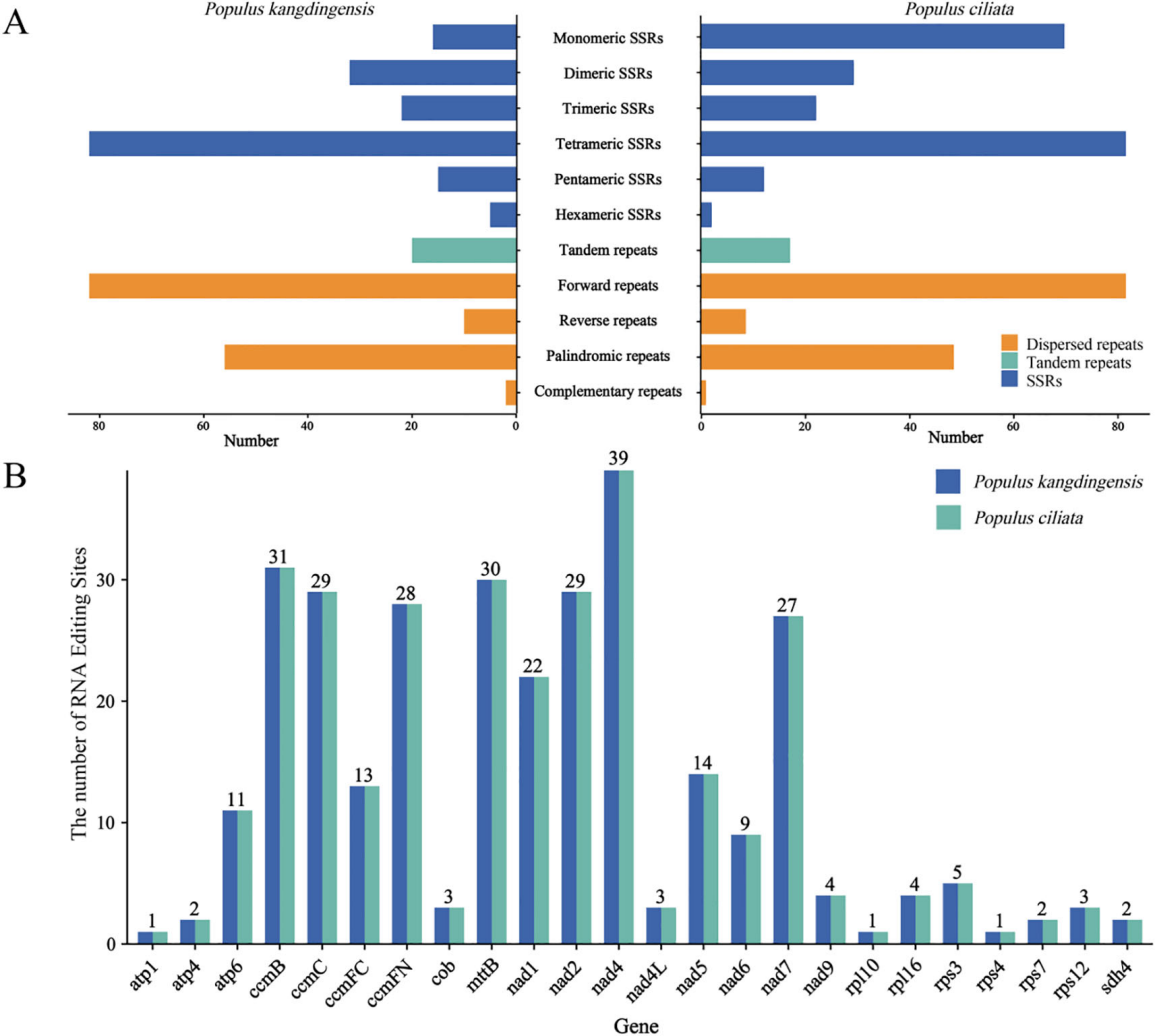
Distribution of repeat sequences and RNA editing site predictions in the mitogenomes of *P. kangdingensis* and *P. ciliata*. **(A)** Integrated distribution of simple sequence repeats (SSRs), tandem repeats, and dispersed repeats across mitochondrial chromosomes. Dispersed repeats are classified into four types: forward (F), palindromic (P), reverse (R), and complement (C). **(B)** Predicted numbers of C-to-U RNA editing sites for each gene. In **(A)** the left side corresponds to *P. kangdingensis* and the right to *P. ciliata*.

Simple sequence repeats (SSRs) are widely distributed in the mitogenome. We identified 227 and 225 SSRs in the mitogenome of *P. kangdingensis* and *P. ciliata*, respectively (**Figure 4A**). Both species exhibited six types of SSR motifs, with similar distribution patterns across types. Notably, no Hexa-type SSRs were detected on the third chromosome in either species. Among these, tetranucleotide repeats were the most abundant, with 82 and 83 loci, accounting for 36.12% and 36.89% of the total SSRs, respectively, and were primarily located on the first chromosome. In contrast, hexanucleotide repeats were the least frequent, with 5 and 3 loci, representing 2.2% and 1.33% of the total SSRs, respectively, and were mainly found on the second chromosome. Tandem repeats were also identified, with 20 and 19 found in *P. kangdingensis* and *P. ciliata* (**Figure 4A**). Both species contained 150 dispersed repeats, including four types: forward (F), palindromic (P), reverse (R), and complement (C), with F and P being the most abundant. The length distribution of dispersed repeats was similar in both species, ranging from 17 to 262 bp and accounting for 84.64% and 84.11% of total repeat length. A total of 313 potential C-to-U RNA editing sites were predicted in each genome (**Figure 4B**). The *nad4* gene exhibited the highest number of editing sites (39), followed by *ccmB* (31). Ribosomal protein genes generally had fewer editing sites.

### 3.5 Analysis of DNA fragment transfer between mitochondrial and chloroplast genomes

**Figure 5:**
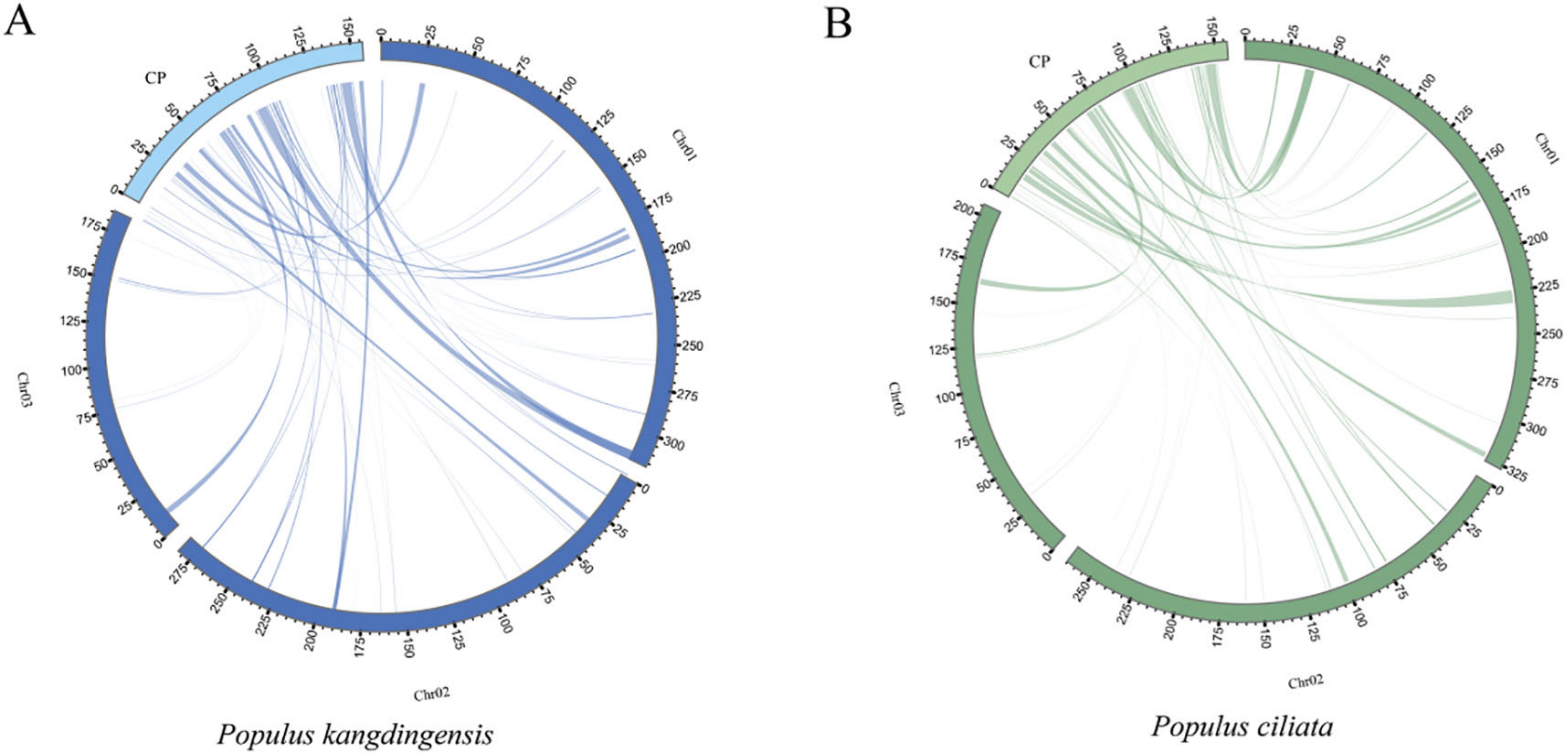
Homologous fragment analysis between the mitochondrial and chloroplast genomes of *P. kangdingensis* and *P. ciliata*. **(A)** Homologous segments between the mitochondrial and chloroplast genomes of *P. kangdingensis*. **(B)** Homologous segments between the mitochondrial and chloroplast genomes of *P. ciliata*. Lines represent plastid-to-mitochondrion transferred fragments (MtPts) identified via sequence similarity. In the circular plots, dark segments indicate mitochondrial genomes and light segments indicate chloroplast genomes.

The transfer of genetic material from chloroplasts to mitochondria is a common phenomenon in the evolution of higher plants. However, these chloroplast-derived sequences often exhibit relatively low conservation. To explore such events in *P. kangdingensis* and *P. ciliata*, we identified homologous fragments between the chloroplast and mitochondrial genomes of both species. A total of 66 homologous fragments were identified in each species through sequence similarity analysis. In *P. kangdingensis*, the homologous fragments range from 29 bp to 2824 bp in length, with a total length of 46,481 bp, accounting for 5.91% of the mitochondrial genome (**Figure 5A**). These fragments display between 0 to 119 mismatches and 0 to 33 gaps (**Supplementary Table S5**). In *P. ciliata*, the fragment lengths ranged from 29 to 3845 bp, with a total length of 48,997 bp, constituting 6.13% of its mitogenome (**Figure 5B**). Mismatches ranged from 0 to 180, and gaps from 0 to 38 (**Supplementary Table S6**). These transferred fragments were termed mitochondrial plastid segments (MtPts). Annotation of MtPts in both species revealed 31 complete genes and 26 partial genes in each genome, suggesting high consistency. However, some differences were observed in gene composition and conservation. Twenty-six complete and twenty-five partial genes were shared between the two species. Unique complete genes in *P. kangdingensis* included *rpl2, rpl23, trnI-CAU, trnL-CAA*, and *trnS-GCU*, while *P. ciliata* had *atpI, rpoC1, rps2*, and fragments of *trnL-CAA* and *trnS-GCU*. Regarding partially overlapping genes, *rpl2* was unique to *P. kangdingensis*, whereas *rpoC1* was specific to *P. ciliata*. A total of 20 tRNA genes were identified: *P. kangdingensis* had 20 completes and 5 partial copies, whereas *P. ciliata* had 18 completes and 5 partial copies. These findings suggest that structural incompleteness or functional degradation of some tRNAs may have occurred during the transfer process. Despite similarities in the number of transferred genes, variations in gene type, integrity, and potential function reflect certain species-specific histories for the retention of exogenous gene fragments in mitochondrial genomes.

### 3.6 Synteny analysis between *P. kangdingensis*, *P. ciliata*, and other species

**Figure 6:**
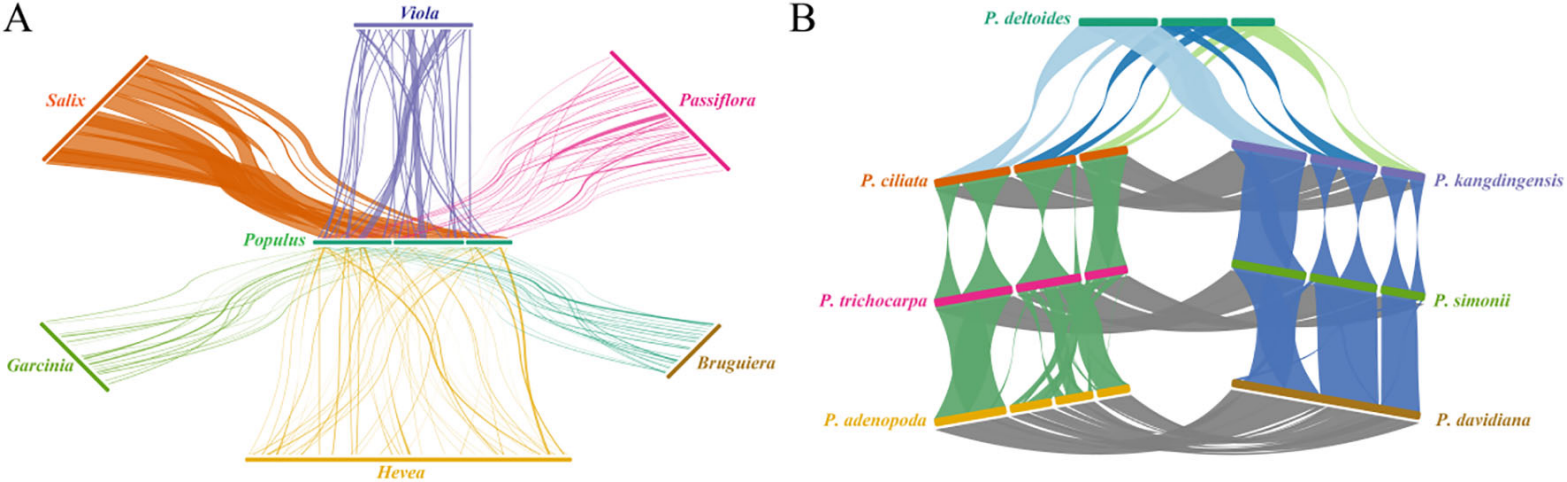
Mitogenome synteny analysis of *P. kangdingensis*, *P. ciliata*, and other species. **(A)** Syntenic relationships between *P. kangdingensis* and six representative species from different genera within Malpighiales. **(B)** Synteny among seven representative species from three *Populus* sections: *Tacamahaca*, *Aigeiros*, and *Populus*. Connecting lines represent syntenic blocks identified between species.

We first examined syntenic relationships between *P. kangdingensis* and six representative species from different genera within Malpighiales (**Figure 6A**). The results revealed significant variability in synteny levels. *P. kangdingensis* and *Salix purpurea* exhibited the highest synteny, with 16 shared syntenic blocks spanning 735,077 bp, accounting for approximately 93.54% of the *P. kangdingensis* mitogenome. In contrast, *Garcinia oblongifolia* shared only 20 syntenic blocks spanning 62,665 bp, representing just 7.97% of the *P. kangdingensis* genome. These differences reflect frequent structural rearrangements in intergenic regions, which contribute to varying degrees of conservation in protein-coding regions across plant mitogenomes. Among these six analyzed species, overlaps between protein-coding regions and syntenic blocks varied significantly (**Supplementary Table S7**), with *S. purpurea* showing the highest overlap (99.76%) and *G. oblongifolia* the lowest (17.28%). These results underscore the structural instability of mitogenomes across species. We further analyzed synteny among seven representative species from three sections within *Populus* (**Figure 6B**). Marked differences in synteny levels were observed among these groups. The highest synteny was between the Sect. *Tacamahaca* and Sect. *Aigeiros: P. ciliata* and *P. kangdingensis* shared syntenic blocks with *P. deltoides* totaling 785,325 bp and 800,598 bp, accounting for 97.84% and 99.75% of the *P. deltoides* mitogenome, respectively. In contrast, synteny between the Sect. *Tacamahaca* and Sect. *Populus* was comparatively weaker, with average synteny lengths of 778,941 bp, covering 98.72% and 97.83% of the *P. alba* and *P. tremula* mitogenomes. Notably, synteny within each section of *Populus* was highly consistent, with coverage ranging from 97.83% to 99.98%, and most protein-coding regions overlapping with syntenic blocks.

### 3.7 Phylogenetic analysis based on chloroplast and mitochondrial genomes in Malpighiales

**Figure 7:**
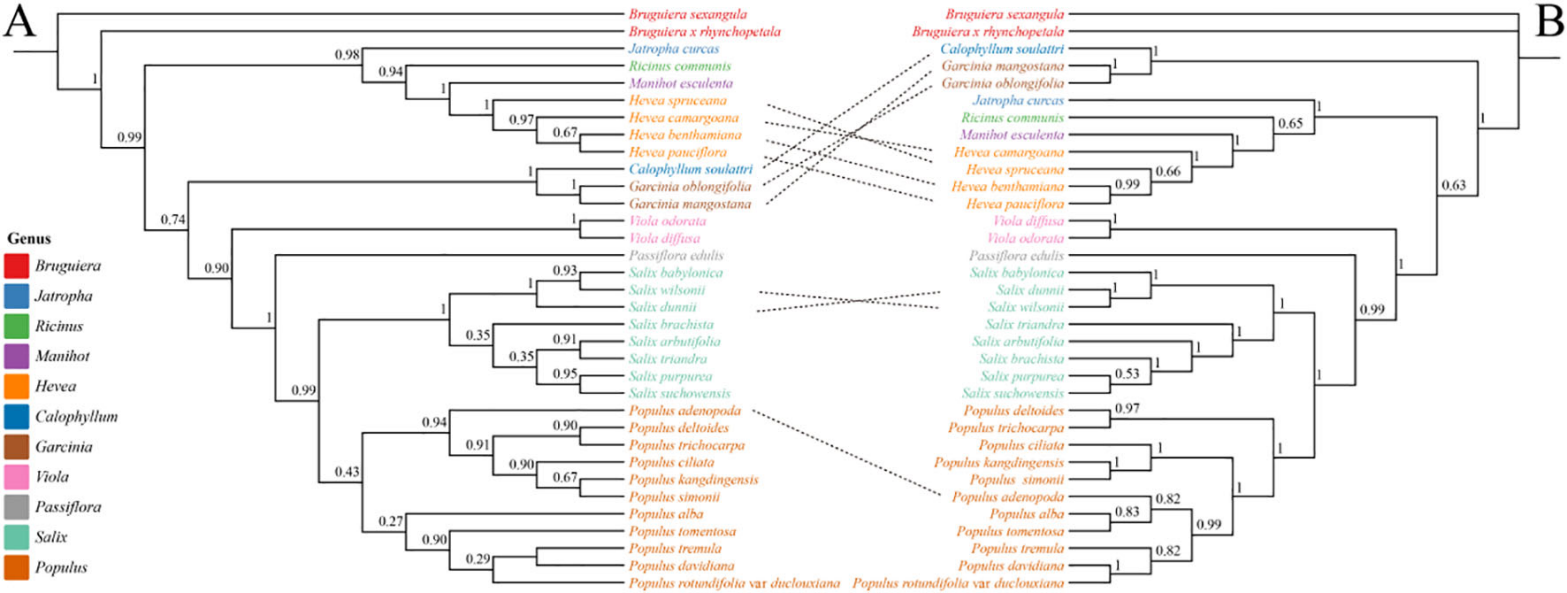
Phylogenetic relationships among different genera of Malpighiales based on chloroplast and mitochondrial genomes. **(A)** Phylogenetic tree constructed using 33 mitochondrial PCGs. **(B)** Phylogenetic tree constructed using 58 shared chloroplast PCGs. The trees include 35 representative species from 11 genera within Malpighiales. Genera are color-coded, and node values indicate bootstrap support levels.

We selected 35 representative species from 11 genera within Malpighiales (*Populus*, *Salix*, *Passiflora*, *Viola*, *Garcinia*, *Calophyllum*, *Hevea*, *Manihot*, *Ricinus*, *Jatropha* and *Bruguiera*) to construct phylogenetic trees using 33 mitochondrial and 58 chloroplast PCGs (**Figures 7A, B**). The two trees showed high topological consistency across most major clades, reflecting well-resolved intergeneric relationships. However, discrepancies in key lineages suggest organellar genomes may capture distinct evolutionary histories. *Populus* and *Salix* were strongly supported as monophyletic sister groups in both trees, indicating a close phylogenetic relationship. However, substantial differences emerged in intra-generic relationships between the two organelle-based trees. In the mitochondrial tree, *P. adenopoda* grouped with *P. kangdingensis*, *P. ciliata*, *P. deltoides*, and *P. trichocarpa*, forming a distinct clade. In contrast, the chloroplast tree placed *P. adenopoda* with *P. alba* and *P. tomentosa* in a clade, which then grouped with *P. davidiana* and *P. tremula*, highlighting discrepancies between mitochondrial and chloroplast phylogenetic signals. Among the complex clade composed of *Hevea*, *Manihot*, *Ricinus*, and *Jatropha*, branching patterns varied between trees. The chloroplast tree supported a stable monophyletic group, while the mitochondrial tree showed a more diffuse topology, with *Hevea* particularly deviating from monophyly. Additionally, *Viola* formed a sister group to the *Hevea*–*Manihot*–*Ricinus*–*Jatropha* clade in the chloroplast tree, but in the mitochondrial tree, it grouped with *Garcinia* and *Calophyllum*. Such conflicts may result from incomplete lineage sorting (ILS), ancient hybridization, or horizontal gene transfer between organelles. Some genera exhibited consistent phylogenetic placements in both trees. *Bruguiera* and *Viola* formed well-supported independent clades across both trees, suggesting minimal impact from conflicting phylogenetic signals. *Garcinia* and *Calophyllum* maintained a stable sister relationship in the chloroplast tree, while in the mitochondrial tree, their positions shifted slightly possibly reflecting differing evolutionary rates or lineage-specific biases in organelle genome history.

## 4 Discussion

### 4.1 Structural diversity and functional conservation in *Populus* mitochondrial genomes

The mitogenomes of higher plants are structurally complex, frequently exhibiting multi-circular, branched, or linear configurations driven primarily by repetitive sequence-mediated recombination (Wu et al., 2022; Cai et al., 2024). Recent studies have also demonstrated that considerable structural differences can exist among mitochondrial genomes within the same genus (Kozik et al., 2019; Wang et al., 2024). In this study, we present the first sequencing, assembly, and characterization of mitogenomes for two representative *Populus* species from the Qinghai–Tibet Plateau: *P. kangdingensis* and *P. ciliata*. The *P. kangdingensis* mitogenome comprises three independent circular molecules, consistent with other species under the *Tacamahaca* section. In contrast, *P. ciliata* exhibits a previously unreported branched mitochondrial genome configuration, composed of two circular molecules and one linear segment distinct from the multi-circular structures described in other *Populus*. Notably, a similar configuration has recently been reported in *Lindera aggregata*, which contains a circular master circle and a linear segment (Shi et al., 2024a), further supporting the structural plausibility of this genome architecture.

Despite differences in genome architecture, both *P. kangdingensis* and *P. ciliata* contain 57 annotated genes, including 34 PCGs, 20 tRNAs, and 3 rRNAs. Notably, the composition of core genes (e.g., *atp, cox, nad, ccm*) is conserved between the two species. This functional conservation has been consistently observed across multiple *Populus* species. Interestingly, the *sdh3* gene is present only in *P. kangdingensis*, *P. ciliata*, and *P. rotundifolia*, while the *rps14* gene is absent in both *P. kangdingensis* and *P. ciliata*,. This phenomen may be attributed to their transfer to the nuclear genome, where they are subsequently expressed (Bonen, 2006; Sloan et al., 2010; Butenko et al., 2024).

### 4.2 Codon usage bias and selection pressure reveal clues to adaptive evolution

Ka/Ks ratio analysis is widely used to evaluate the selective pressures acting on genes during evolution and provides insights into species-specific adaptive changes and the direction of natural selection. Here, Ka/Ks analysis of mitochondrial PCGs in ten *Populus* species revealed that most genes are under strong purifying selection, reflecting the evolutionary conservation of protein function. This pattern is common among higher plants and suggests the structural and functional stability of key respiratory and energy metabolism enzyme complexes (Mower et al., 2012). Notably, a subset of genes (*atp4, ccmB, ccmFN, and mttB*) provide the evidence of positive selection across the ten species. As mitochondria play a central role in energy metabolism, these genes may be subject to directional selection under specific environmental conditions, potentially enhancing species’ adaptability and metabolic flexibility (Wang et al., 2021). (Lyu et al., 2024). conducted a selection analysis of mitochondrial genes across 58 species from different altitudes using the aBSREL model and identified three candidate genes potentially involved in high-altitude adaptation: *atp4*, *atp9*, and *mttB*. Among them, *atp4* encodes a critical subunit of ATP synthase (Heazlewood et al., 2003), which may play a role in plant growth and development. In several high-altitude plant species, numerous transcriptional elements have been identified in the vicinity of *mttB*, which are likely induced by environmental pressures associated with high-altitude adaptation, suggesting a possible role of *mttB* in this process (Xiong et al., 2022). We analyzed the expression levels of 32 PCGs from the mitochondrial genomes of two *Populus*. The results showed that *atp4* and *mttB* exhibited higher expression in *P. kangdingensis*, which is distributed at higher elevations (**Supplementary Figure S5**). Therefore, *atp4* and *mttB* may be candidate genes for high-altitude adaptation in the mitochondrial genome of *Populus* and warrant further investigation.

Codons play a central role in the transmission of genetic information and protein synthesis. Due to factors such as gene mutation and natural selection, codon usage bias has emerged as an important evolutionary signature of genome evolution and selection pressure (Wang et al., 2018; Yengkhom et al., 2019). In this study, we systematically analyzed codon usage patterns for 34 PCGs in the mito genomes of *P. kangdingensis* and *P. ciliata*. Our results indicate a strong preference for A/T bases and A/T-ending codons in both species, consistent with the reported A/T bias in the *P. deltoides* mitogenome. This AT-rich tendency at the third codon position is a common feature in higher plants (Qu et al., 2023).

Further ENC-plot analysis revealed that most genes were located far from the theoretical curve, suggesting that codon usage is non-random and significantly associated with gene expression levels, implying strong natural selection. This trend aligns with findings in model plants such as *Arabidopsis*, rice, and wheat, supporting the view that natural selection is a key driver of codon usage bias (Sharp and Li, 1987; Wang and Hickey, 2007). Our findings suggest that codon usage bias in *Populus* mitochondrial genomes may reflect both functional constraints in coding regions and adaptive evolutionary patterns.

### 4.3 Species-specific differences in mitochondrial RNA editing and chloroplast-to-mitochondrion DNA transfer

RNA editing is a crucial post-transcriptional regulatory mechanism in plant mitochondrial genomes, primarily involving C-to-U conversions, which can alter amino acid codons and thus affect protein function (Hao et al., 2021). In both *P. kangdingensis* and *P. ciliata*, 313 RNA editing sites were detected, with genes such as *nad4* and *ccmB* harboring the highest number of sites. These findings are consistent with results from species in the *Populus* section, such as *P. tremula* and *P. alba*, suggesting a central role for these genes in mitochondrial functional regulation (Sung et al., 2010). Conversely, *rps* exhibited fewer editing events, likely reflecting their functional conservation, which may negate the need for optimization through editing.

Transfer of genetic fragments from the chloroplast to the mitochondrion is a common feature of plant organelle genome evolution and has been observed across many plant taxa (Gualberto et al., 2014). In this study, we identified 66 plastid-derived transfer fragments (MtPts) in *P. kangdingensis* and *P. ciliata*, accounting for 5.91% and 6.13% of their mitochondrial genomes, respectively. These values are higher than those reported in the *Aigeiros* section species *P. deltoides* (4.11%) and in the *Populus* section species *P. tomentosa* (4.42%), and the fragments include both complete and partial tRNAs and PCGs. Since *P. kangdingensis* and *P. ciliata* belong to the more derived Sect. *Tacamahaca* compared to Sect. *Populus*, this observation may suggest an evolutionary trend toward increasing chloroplast-derived tRNA integration into mitochondrial genomes (Glover et al., 2001).

Although the two species share 31 identical transferred genes, most of these gene transfers are non-functional. For instance, the transferred gene *rpl2* in *P. kangdingensis* is unique to this species and corresponds to its mitochondrial *nad5* gene, while the species-specific transferred gene *rpoC1* in *P. ciliata* is non-functional. Transcriptome data further reveal that only a subset of chloroplast-to-mitochondrion transferred genes (*atp1, nad1, rps1, and nad5*) are expressed in both species. This may reflect species-specific mechanisms of gene transfer and selective retention (Hertle et al., 2021; Kelly, 2021). These observations support the hypothesis that mitochondria exhibit a “selective acceptance” of foreign DNA fragments (Timmis et al., 2004; Smith and Keeling, 2015).

### 4.4 Phylogenetic topology conflicts reveal inconsistencies in lineage history

Using *P. kangdingensis* as a representative, we performed a comprehensive synteny analysis between its mitochondrial genome and those of six other genera within *Malpighiales*. Our results revealed substantial differences in synteny levels among genera. The highest synteny was observed between *Populus* and *Salix*, covering nearly the entire set of protein-coding regions; however, *Garcinia* exhibited the lowest synteny, suggesting high structural conservation in Salicaceae and a closer evolutionary relationship between its members. In contrast, the structural divergence observed between more distantly related genera may result from mechanisms such as recombination mediated by repeats and DNA loss (Gualberto and Newton, 2017).

Within *Populus*, differences in synteny levels were also observed between sections. The Sect. *Tacamahaca* and *Aigeiros* exhibited higher synteny compared to the *Populus* section. Nevertheless, all seven examined *Populus* species showed synteny levels exceeding 97%, indicating substantial structural conservation across the genus during mitochondrial genome evolution.

Both plastid phylogenetic trees strongly support the monophyly of *Populus* and *Salix*, identifying them as sister groups, a result consistent with the classification of Salicaceae proposed by the APG IV system (The Angiosperm Phylogeny Group et al., 2016). *P. kangdingensis*, *P. ciliata*, and *P. simonii* form a monophyletic clade, and the topologies of the two plastid phylogenies align with those of the nuclear gene tree for *Populus* on the Qinghai-Tibet Plateau reported by Mi (Mi et al., 2025), displaying high support values.

However, in the mitochondrial phylogeny, *P. adenopoda* (a member of the Sect. *Populus*) does not group with other Sect. *Populus* taxa as it does in the plastid phylogeny, but instead forms a clade with members of the Sect. *Tacamahaca* and Sect. *Aigeiros*. This result is inconsistent with the classification of *Populus* in the Flora of China. Previous studies (Wang et al., 2022) using nuclear gene trees showed that *P. adenopoda* clusters with other Sect. *Populus* species, in agreement with the plastid phylogeny in this study. Such conflict may be attributed to widespread introgression and incomplete lineage sorting (ILS) within *Populus* (Wang et al., 2020).

Overall, while these topological differences largely align with the APG IV framework at the intergeneric level, the species-level discrepancies within the genus underscore the complementary yet distinct evolutionary signals from organellar and nuclear phylogenies (Degnan and Rosenberg, 2009). Therefore, integrating mitochondrial, plastid, and nuclear genomic data is crucial for reconstructing a more accurate evolutionary history and phylogenetic relationships among species.

## 5 Conclusion

This study firstly reports the high-quality mitochondrial genome assemblies of *P. kangdingensis* and *P. ciliata* from the Qinghai-Tibet Plateau. The two species exhibit distinct genome structures multi-circular in *P. kangdingensis* and branched in *P. ciliata*—highlighting structural diversity within *Populus*. Both genomes contain 57 conserved functional genes, but show variation in intron distribution, repeat content, and codon usage bias. Widespread RNA editing and substantial chloroplast-to-mitochondrion DNA transfer were also observed. Phylogenetic analyses support the monophyly of *Populus* and *Salix*, but reveal discordance among other genera, suggesting organelle-specific evolutionary histories. These results provide valuable genomic resources and insights into the evolution and adaptation of *Populus* species in high-altitude environments.

## Data Availability

The mitochondrial genomes of *P. kangdingensis* have been deposited in GenBank under accession numbers PX116244, PX116245, and PX116246, while those of *P. ciliata* are available under accession numbers PX116247, PX116248, and PX116249. The raw sequence data have been deposited in the Genome Sequence Archive in National Genomics Data Center, China National Center for Bioinformation / Beijing Institute of Genomics, Chinese Academy of Sciences (GSA: CRA028946) that are publicly accessible at https://ngdc.cncb.ac.cn/gsa/browse/CRA028946.
