# Supplementary material for: Structural diversity and phylogenomic insights from the mitochondrial genomes of two populus species from the Qinghai-Tibet Plateau
Source: Front Plant Sci. 2025 Sep 12;16:1637726. doi: 10.3389/fpls.2025.1637726 (PMC12471105; doi:10.3389/fpls.2025.1637726)
Supplement: Supplementary file 1 [file DataSheet1.docx]

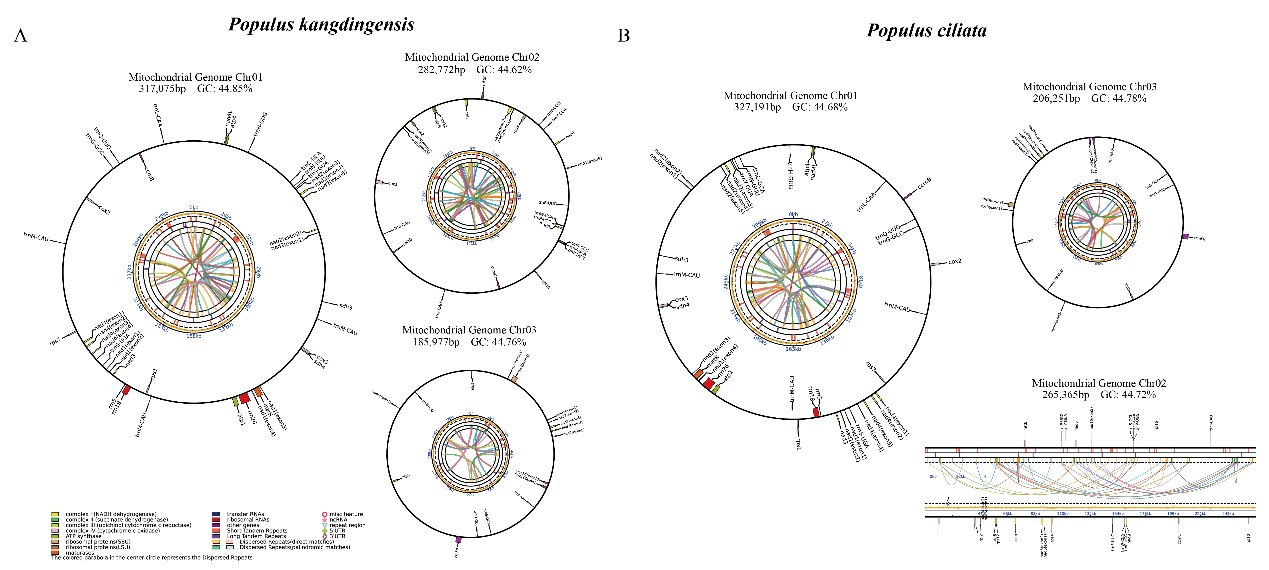


Fig S1. Structural representation and annotation of the mitochondrial genomes of P. kangdingensis and P. ciliata. (A) Annotated map of the mitochondrial genome of P. kangdingensis. (B) Annotated map of the mitochondrial genome of P. ciliata. Genes are color-coded by functional category: protein-coding genes (PCGs), transfer RNAs (tRNAs), and ribosomal RNAs (rRNAs). Dispersed repeats are illustrated as colored arcs indicating homologous regions either within or between molecules.


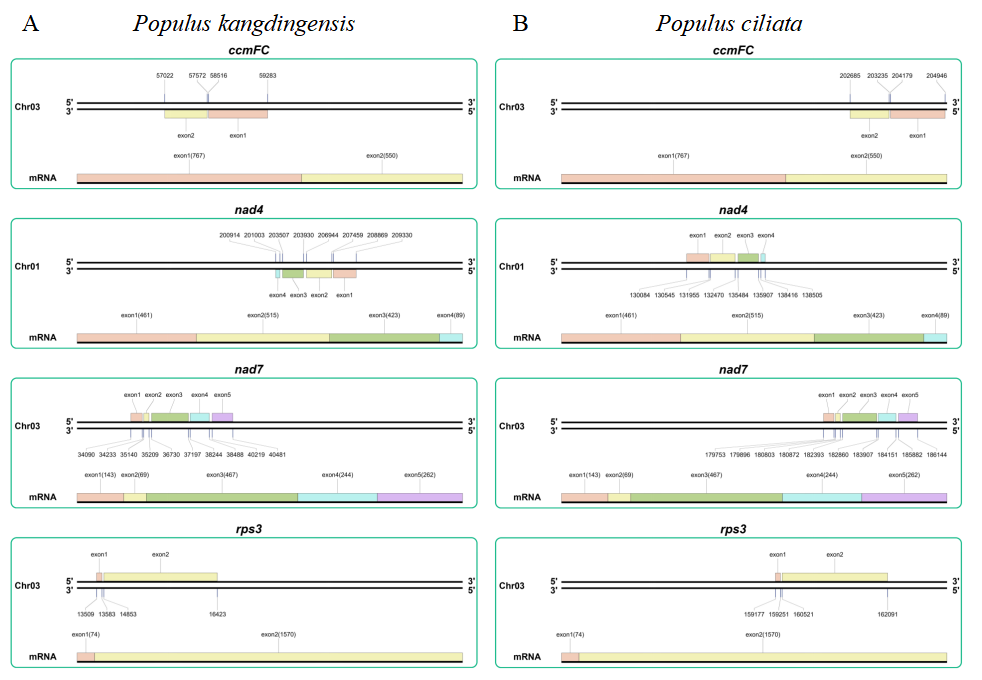


Fig S2. Identification of cis-splicing genes in the mitochondrial genome of *P. kangdingensis* and *P. ciliate*.


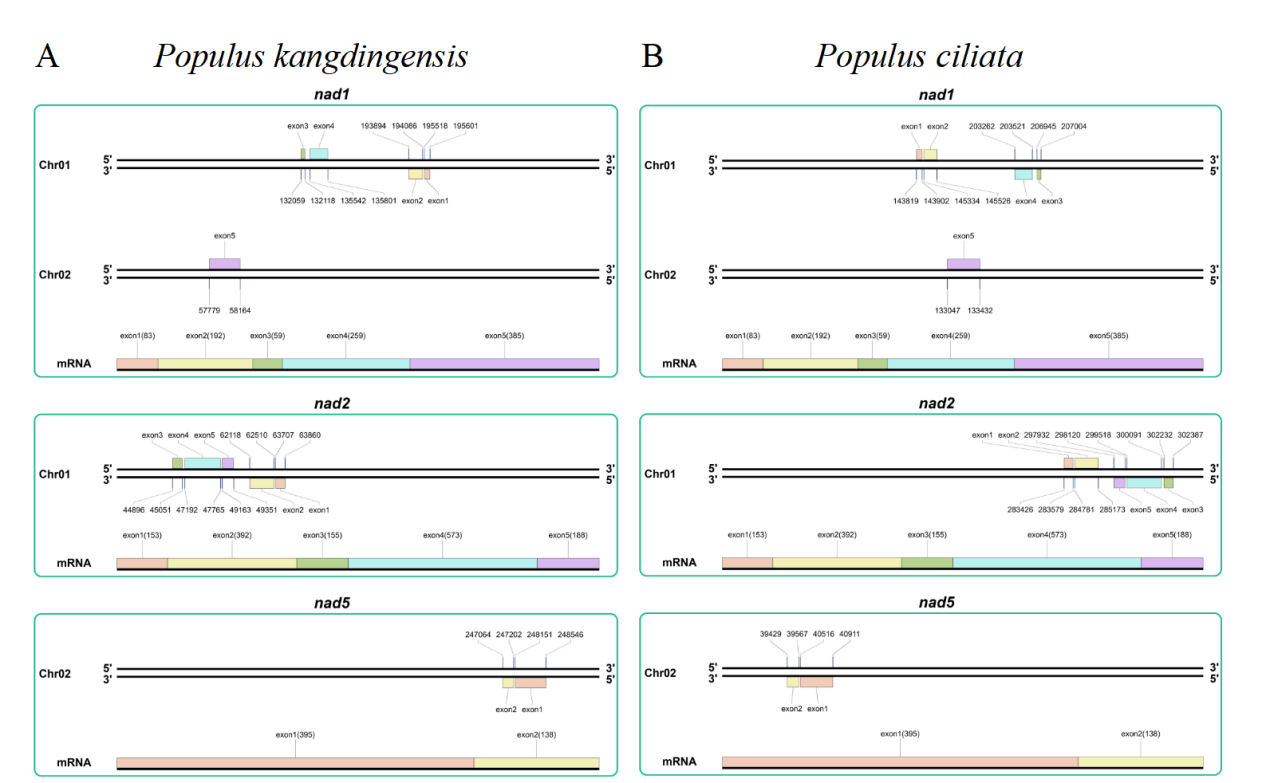


Fig S3. Identification trans-splicing genes (B) in the mitochondrial genome of *P. kangdingensis* and *P. ciliate*.


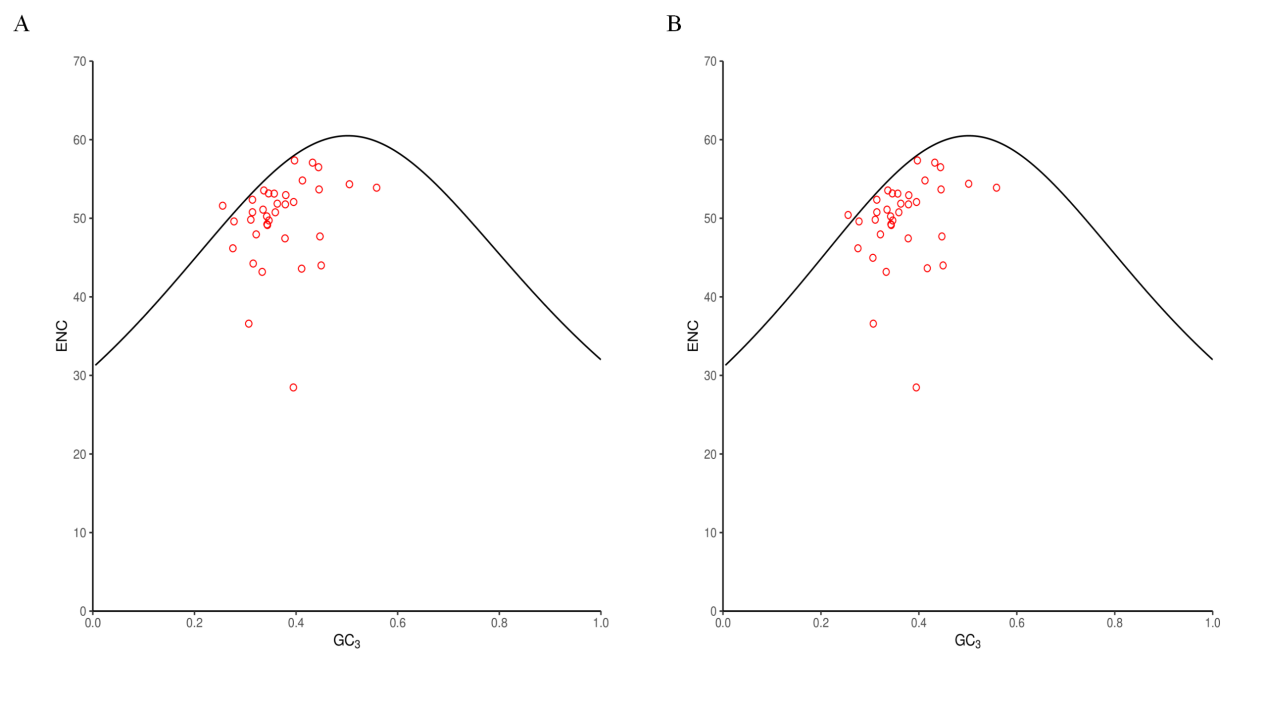


Fig S4. Ranges of ENC content (%) among 34 PCGs in P. kangdingensis and P. ciliate.


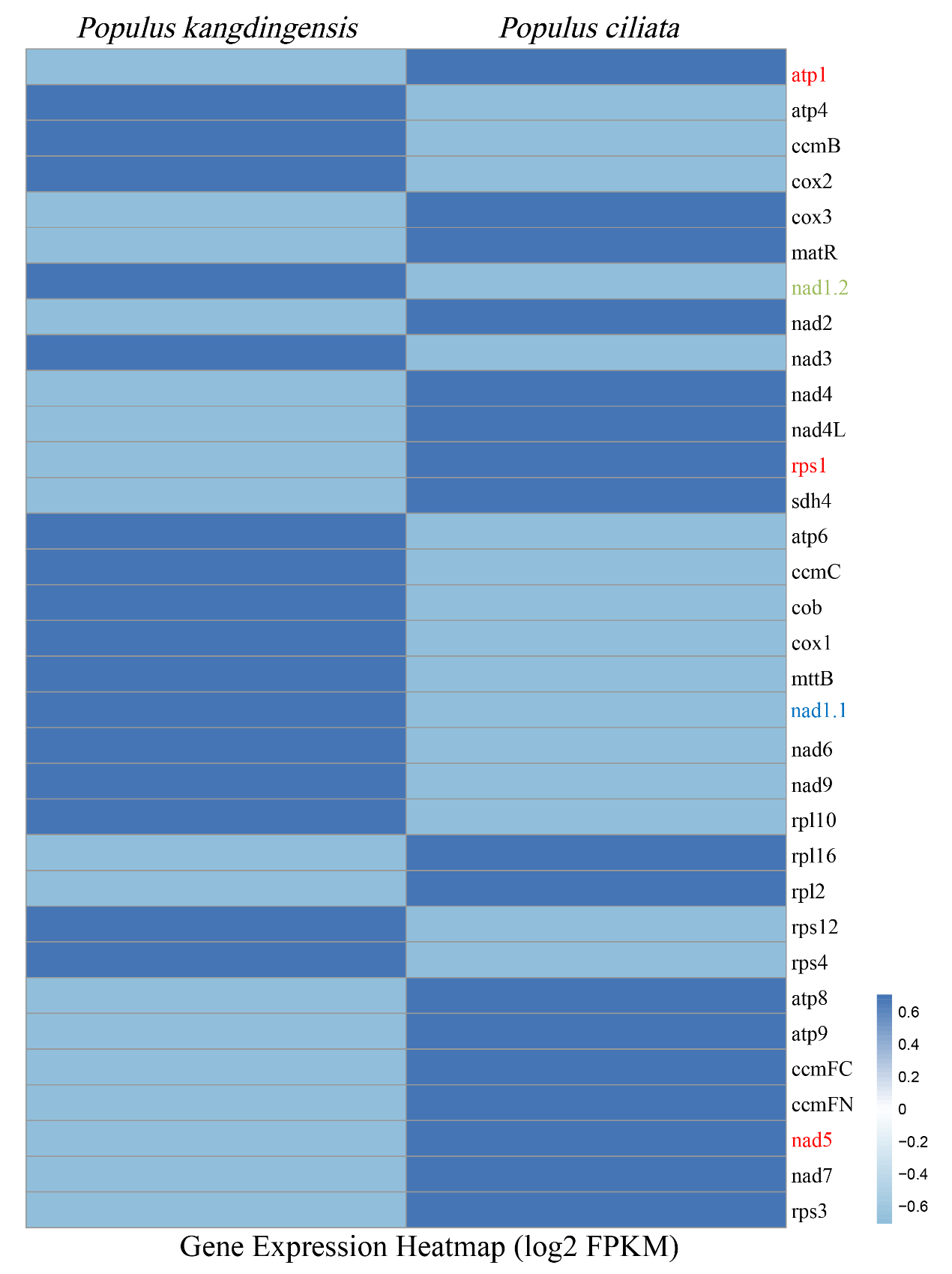


Fig S5. The heatmap shows the transcriptomic expression levels of 32 mitochondrial protein-coding genes (PCGs) in *P. kangdingensis* and *P. cliata*. Genes highlighted in red represent chloroplast-derived sequences shared by both species; those in blue are unique chloroplast-to-mitochondrion transfers found only in *P. kangdingensis*, while those in green are unique to *P. cliata*.
